# Development of Electrochemical Aptasensor for Lung Cancer Diagnostics in Human Blood

**DOI:** 10.3390/s21237851

**Published:** 2021-11-25

**Authors:** Anastasiia V. Shabalina, Darya O. Sharko, Yury E. Glazyrin, Elena A. Bolshevich, Oksana V. Dubinina, Anastasiia M. Kim, Dmitry V. Veprintsev, Ivan N. Lapin, Galina S. Zamay, Alexey V. Krat, Sergey S. Zamay, Valery A. Svetlichnyi, Anna S. Kichkailo, Maxim V. Berezovski

**Affiliations:** 1 Siberian Physical-Technical Institute, Tomsk State University, 634050 Tomsk, Russia; shabalinaav@gmail.com (A.V.S.); daryasharko29@gmail.com (D.O.S.); elena17051305.ya.ru@gmail.com (E.A.B.); kamchatuska@rambler.ru (O.V.D.); evdokimova@k21.center (A.M.K.); 201kiop@mail.ru (I.N.L.); v_svetlichnyi@bk.ru (V.A.S.); 2 Federal Research Center, Krasnoyarsk Science Center of the Siberian Branch of the Russian Academy of Science, 660036 Krasnoyarsk, Russia; yury.glazyrin@gmail.com (Y.E.G.); d_veprintsev@mail.ru (D.V.V.); galina.zamay@gmail.com (G.S.Z.); sergey-zamay@yandex.ru (S.S.Z.); 3 Laboratory of Biomolecular and Medical Technologies, Krasnoyarsk State Medical University Named after Prof. V.F. Voyno-Yasenetsky, 660022 Krasnoyarsk, Russia; alexkrat@mail.ru; 4 Krasnoyarsk Regional Clinical Cancer Center Named after A.I. Kryzhanovsky, 660133 Krasnoyarsk, Russia; 5 Department of Chemistry and Biomolecular Sciences, University of Ottawa, Ottawa, AB K1N 6N5, Canada

**Keywords:** electrochemical aptasensor, biosensing layer, useful signal, lung cancer, human blood

## Abstract

We describe the preparation and characterization of an aptamer-based electrochemical sensor to lung cancer tumor markers in human blood. The highly reproducible aptamer sensing layer with a high density (up to 70% coverage) on the gold electrode was made. Electrochemical methods and confocal laser scanning microscopy were used to study the stability of the aptamer layer structure and binding ability. A new blocking agent, a thiolated oligonucleotide with an unrelated sequence, was applied to fill the aptamer layer’s defects. Electrochemical aptasensor signal processing was enhanced using deep learning and computer simulation of the experimental data array. It was found that the combinations (coupled and tripled) of cyclic voltammogram features allowed for distinguishing between the samples from lung cancer patients and healthy candidates with a mean accuracy of 0.73. The capacitive component from the non-Faradic electrochemical impedance spectroscopy data indicated the tumor marker’s presence in a sample. These findings allowed for the creation of highly informative aptasensors for early lung cancer diagnostics.

## 1. Introduction

Electrochemical aptasensors are becoming popular in sensing a wide range of different analytes, from cells [1] and microorganisms [2], to small molecule biomarkers [3,4,5] and metal ions [6,7,8] in body fluids (serum [9,10], urine [11], and saliva [12]), food [13,14,15], and water [16,17]. When increasing the sensitivity and other analytical characteristics of electrochemical aptasensors, a tendency to complicate their composition and structure is observed. Such complications include an addition to the aptasensor of nanoparticles [18,19], as well as graphite and carbonaceous structures [20,21,22], using two or more aptamers, sandwich structures, “competitive assays” [23,24], and additional antibodies and enzymes [25,26]. Only 10% of published articles have dealt with a simple setup consisting of an aptamer recognizing layer on the electrode surface (for example [27]). The scheme of generating a signal from the aptasensor is also becoming more and more complicated. In the literature, there are examples of using additional light irradiation [28,29,30], signal amplification due to additional components (nanoparticles, graphene, etc.) [31,32,33], ratiometric schemes [34], and so on. However, the more complex the system, the less reproducible and stable it is when in use [30]. Furthermore, an improved understanding of the key factors in the origin and response formation of the biosensor signal is an important step towards developing advanced electrochemical sensing platforms [35]. Generally, the electrochemical responses of the aptasensors in the presence and absence of target molecules are compared, and known hypothetical mechanisms are suggested [36]. We propose a detailed study considering the performance of the mechanism of the formation of an aptasensor’s electrochemical response using a simple aptamer/electrode system (without additional complications).

Thiolated aptamer layers on a gold electrode (or gold particles’ surface, etc.) is a classic approach and is still quite a popular basis for electrochemical aptasensors [37]. The choice of a better gold electrode pretreatment was an interesting challenge [38,39]. In this work, we first chose a reductive pretreatment to prepare an aptasensor based on the DNA aptamer LC-18 developed earlier for postoperative lung cancer tissues [40]. This aptamer has already proven its ability to bind to plasma lung cancer biomarkers [41], detect circulating tumor cells in blood [40], and selectively label cancer cells in lung tumors [41]. Lamin, vimentin [41], histone H2B, and neutrophil defensins 1 and 3 [42] are the biomarkers associated with LC-18. These proteins play a role in human malignancy [41,43,44] and are overexpressed in several tumor types [45,46,47]. Cells’ malignant transformations can be caused by nucleosomes dysfunction and impaired gene expression [48]. Histone H2B regulates nucleosomes stability, chromatin availability, remodeling, mutations, and post-translational modifications. Neutrophil defensins, the main components of neutrophils azurophilic granules, are involved in various cellular processes, including cell proliferation. These peptides are believed to have an antitumor activity [49]. They can be presented in a natural conformation in the blood plasma of lung cancer patients in an amount that is sufficient for detection. To develop a new test system prototype, the detecting capabilities of these proteins in natural concentrations in plasma were tested using an aptasensor with an LC-18 aptamer layer.

In addition, the first attempts to study the stability of the composition, structure, and binding ability of the LC-18 aptamer layer were made.

A blocking agent (BA) is used to fill the electrode surface uncovered with the aptamer. In this work, we, for the first time, suggested blocking the uncovered electrode surface with a new blocking agent—a short thiolated oligonucleotide with an unrelated sequence. This blocking oligonucleotide has the same length as the linker binding the aptamer with gold. So, it fills the defects in the aptamer layer and does not hinder aptamer molecules from binding the target object because its length is sufficiently short. Moreover, this blocking agent’s nature is close to the aptamer, so additional unwanted intermolecular interactions would not happen.

Thus, this work includes, first of all, the preparation of the sensing layer of the LC-18 aptamer specific to the lung cancer tumor marker on the gold electrode. The aptamer layer’s structure stability and binding ability were studied using electrochemical methods and confocal laser microscopy. New BA was suggested and tested, enhancing the effectiveness of the signal processing of the electrochemical aptasensor using deep learning and computer simulation of the experimental data array.

## 2. Materials and Methods

### 2.1. Reagents

All of the reagents listed below were obtained from Merck and Alfa Aesar (Thermo Fisher Scientific, Waltham, MA, USA) and were used as received. The following substances were utilized in the present work: KCl, NaOH, H_2_SO_4_, H_2_O_2_, NaBH_4_, CaCl_2_, MgCl_2_, K_3_[FeCN_6_], K_4_[FeCN_6_], phosphate buffer tablets, 1-dodecanethiol, and acetone. MilliQ or distilled (where possible) water was used.

### 2.2. Oligonucleotides

Oligonucleotides were synthesized in the following (Integrated DNA Technologies, Coralville, IA, USA): blocking agent in the form of a blocking thiolated oligonucleotide (BTO, 5′-SH-(CH_2_)_6_-GGG AGG AGA CTG ACA TTG GTG CA-3′), 5′-thiolated DNA-aptamer LC-18 (5′-SH-(CH_2_)_6_-CTCCTCTGACTGTAACCACGTGCCCGAACGCGAGTTGAGTTCCGAGAGCT CCGACTTCTT GCATAGGTAG TCCAGAAGCC-3′) with a high specificity to lung cancer cells [40], tissues [41], and tumor markers in the blood plasma [42].

### 2.3. Aptasensor Preparation

#### 2.3.1. Gold Electrode Pretreatment

Aptasensors were prepared based on gold disc electrodes of 2 mm in diameter. The electrodes were pretreated before the aptamer immobilization. The pretreatment included three main stages. (1) Mechanical treatment (abrasion and polishing) and ultrasonication in water and ethanol for 10 min each. (2) Chemical etching in a Piranha solution (a mixture of concentrated solutions: 3 mL of H_2_SO_4_ and 1 mL of H_2_O_2_) for 5 min with the subsequent drying under Ar flow. (3) Electrochemical treatment. The method of electrochemical treatment was chosen based on the experiments with five different reductive techniques listed in Table 1. A chemical reduction with NaBH_4_ was also applied for comparison.

After the third stage, the electrodes were immediately immersed in the aptamer solution.

To estimate the surface coverage ability of the pretreated electrodes, 1-dodecanethiol (1-DDT or DDT) was used. The electrodes were dipped into DDT 1% solution in ethanol for 1, 4, 16, and 24 h. The surface coverage was calculated as the percentage ratio of the redox probe oxidation peaks before and after DDT immobilization.

#### 2.3.2. Aptamer Immobilization

An aptamer solution (1 or 10 µM) in PBS (with Ca^2+^ and Mg^2+^ ions) was used to immobilize the aptamer molecules on the electrode. Electrodes after pretreatment were immersed in the solution and kept for 16 h at 4 °C and 100% relative humidity. Then, the electrodes were thoroughly rinsed with PBS to remove excess aptamer in the solution and the non-specifically bound molecules on the surface. After that, the electrodes were put into an additional blocking agent solution (a blocking thiolated oligonucleotide, BTO, see Section 2.2.) for 6 h (4 °C, 100% RH) and then rinsed with PBS for the experiments with blood plasma. Before and between the measurements, aptasensors were kept in PBS solution at 4 °C.

### 2.4. Aptasensor Characterization

#### 2.4.1. Electrochemical Characterization

Cycling voltammetry (CV) and electrochemical impedance spectroscopy (EIS) were used to characterize the layer of biomolecules on the electrode surface. The data were registered using the electrochemical workstation CH-660E (CH Instruments, Austin, TX, USA) using a three-electrode sealed glass cell with Pt plate counter and Ag/AgCl (1 M KCl) reference electrodes. Phosphate buffer solution (PBS) with a pH of 7.4 was used as a background electrolyte. An equimolar solution (0.025 M) of both potassium hexacyanoferrates (II and III) in PBS was used for the experiments with a redox probe. All of the solutions were preliminarily degassed with Ar (30 min), and then before each measurement, 5 min of Ar purging was additionally applied.

CVs were collected in the potential range of −0.5 to 0.8 V with a scan rate of 0.03 V/s. Impedance spectra were registered in the frequency range from 1 Hz to 1 kHz with the potential amplitude of 0.01 V. Initial potential was established at open circuit potential (E_OC_) for measurements in PBS and at E1/2 for measurements in the redox probe solution (E_1/2_ = 0.5 × (Ea + Ec), where Ea and Ec are potentials of anodic and cathodic peaks, respectively). A range of 3 to 10 measurements were carried out for every parallel experiment.

Surface coverage was determined by the peak area of the redox probe oxidation from CVs, according to the Randles−Sevcik equation.

#### 2.4.2. Microscopic Characterization

The structure of the biomolecules’ layer on the electrode was characterized by confocal laser scanning microscopy (CLSM). An inverted-scheme microscope LSM 780 NLO (Carl Zeiss, Germany) was used. The objectives of 20× and 100 × (oil) were applied for the image collecting. The 3D images of the surface layer were obtained using a z-stag mode with a 3D reconstruction.

### 2.5. Blood Plasma Samples Analysis

All of the experiments were carried out in accordance with the approved guidelines and principles expressed in the Declaration of Helsinki. The experimental protocol was approved by the Local Committee on Ethics of the Krasnoyarsk Regional Clinical Cancer Center, named after A.I. Kryzhanovsky No. 8/2011 since 16 March 2011, and Krasnoyarsk State Medical University 37/2012 since 31 January 2012, Krasnoyarsk, Russia. Informed consent was secured from all patients in this study. Blood samples were taken from 10 patients with morphologically verified lung cancer and without metastasis before complete tumor resection and therapy. Five patients had squamous lung cancer and five patients had adenocarcinoma. Control samples were taken from 10 healthy individuals. Age and gender were not taken into account. Blood samples were collected using vacutainers with heparin and were processed within two hours of collection.

Plasma was obtained by centrifugation at 3000 rpm for 30 min and was used fresh or stored at 4 °C no longer than 24 h prior to the experiments. The plasma samples were diluted two times with PBS and incubated with yeast RNA (at a final concentration of 1 nM) for 30 min on a shaker to mask non-specific DNA binding sites.

#### 2.5.1. Immobilization of the Target Protein from Plasma

The electrode, previously prepared according to Section 2.3.2 and passed the measurement procedure according to Section 2.5.1, was incubated with plasma (diluted two times with BPS solution) for 30 min at 4 °C.

#### 2.5.2. Electrochemical Response from the Aptasensor

**Figure 1:**
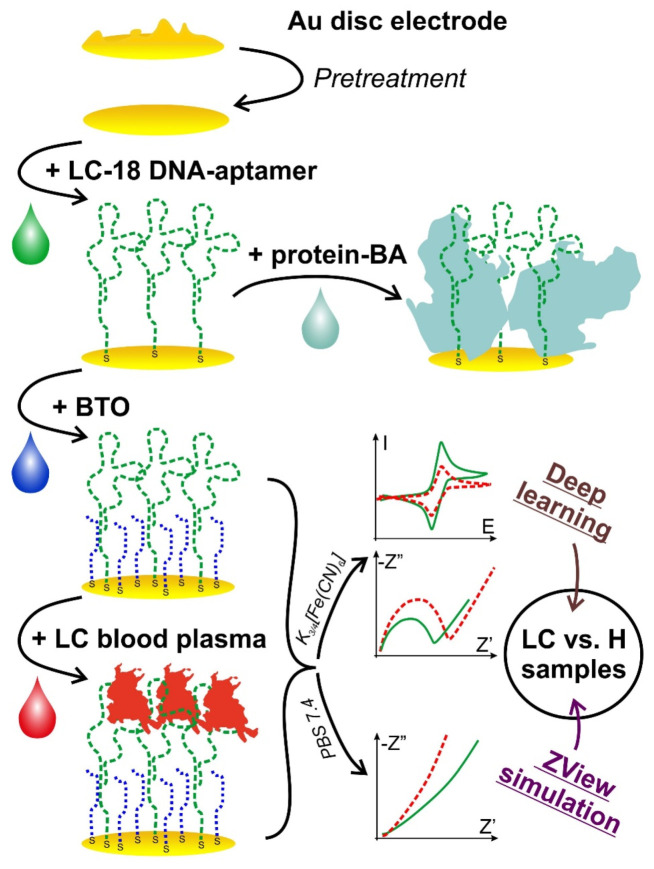
A schematic representation of the aptasensor preparation and study process. Top-down: electrode pretreatment stage; aptamer immobilization; protein BA (to the right) or BTO that is an oligo-BA (downward) immobilization; incubation with the blood plasma; electrochemical measurements in two solutions and data processing (to the right).

The electrochemical responses of the aptasensor (CVs and EIS) were recorded according to 2.4.1 (Figure 1) before and after its incubation with the blood plasma sample.

### 2.6. Data Processing

The CV and EIS data for each sample were transformed into a set of numerical features. Furtermire, we added class information “LC”/“H” (“lung cancer”/“healthy”). The relationship between features and class as a classification problem was studied. The whole analysis was performed using Anaconda Python 3.6. EIS models were simulated using ZView software (Scribner Associates Inc.; Southern Pines, NC, USA). The models obtained were fitted with the experimental data, and the parameters of equivalent circuits were calculated.

## 3. Results and Discussion

### 3.1. The Procedure of the Development of Electrochemical Aptasensor for Real Biological Samples Analysis

Here, we present the development of the electrochemical aptasensor for analyzing cancer biomarkers in a real blood plasma without preliminary experiments on the isolated target protein–tumor marker. The LC-18 aptamer has already demonstrated its ability to identify lung cancer-related proteins and cells in patients and tissues [40,41,42]. Therefore, it was chosen for the aptasensor development. We focused on the difference between the responses from the blood plasma of LC patients and healthy candidates. Distinguishing these responses means that the LC-18 aptamer binds to the tumor markers in the LC samples, and their presence can be detected.

### 3.2. Biosensing Layer Preparation and Study

Mi-Na et al. [50] compared aptamer layers bound to the Au surface through thiol and amino groups and showed that the Au-S bond was preferred. Self-assembled monolayers (SAMs) of thiols on the gold surface have been studied extensively for more than 25 years. The SAMs of alkanethiols, hydroxy thiols, and other thiolated organics are easy to obtain and study. They can form quite dense layers with a high surface coverage. The structure, defectiveness, and dynamics of these SAMs, as well as electron tunneling through them, were studied [51,52]. Aptamer molecules, being much larger and having a more complicated structure, exhibited a different behavior when forming SAMs. Some results obtained for SAMs of single- and double-stranded DNA (ssDNA and dsDNA, respectively) on electrodes [53,54] might be applied for the DNA-aptamer layer description. Each aptasensor needs to be optimized individually [55].

The choice of a better gold electrode pretreatment was an interesting challenge [38,39]. However, different pretreatments might be required for different aptamer’s SAM formation because of their molecules’ different composition and structure [56]. So, the best electrode pretreatment choice is a step of the aptasensor development process. The most widely used pretreatment protocol includes polishing, etching with a Piranha solution, and electrochemical treatment. The latter is very often performed in the H_2_SO_4_ solution and the region of positive potential. However, the gold oxide thin layer and oxide species on the electrode surface might hinder the Au-S bond formation [51]. So, reductive treatment is quite desired for successful SAM formation [56,57,58].

#### 3.2.1. Electrode Pretreatment Procedure Development

**Figure 2:**
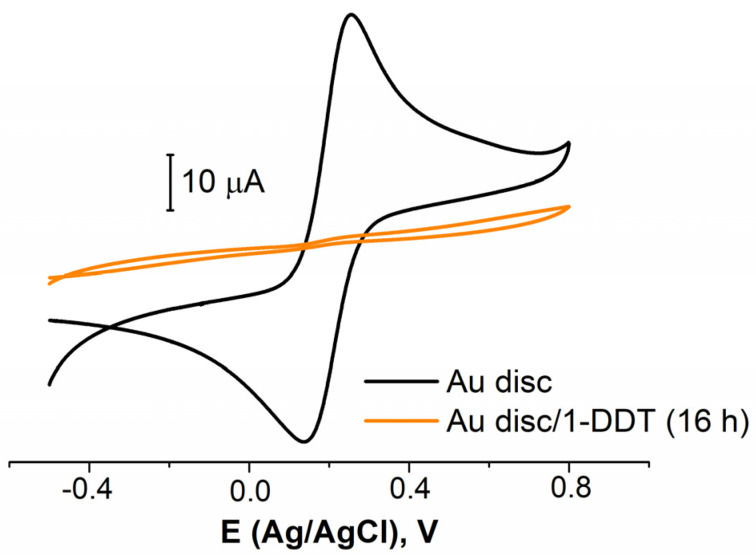
CVs for bare Au disc electrode (black curve) and covered with 1-DDT (colored curve). Solution: 0.025 M K_3/4_[Fe(CN)_6_] in PBS (7.4).

Five different electrochemical and one reductive chemical pretreatments were studied (Table 1, rows 1−6). The 1-dodecanthiol (1-DDT or DDT) was used to evaluate the electrode coverage as a model thiolated organic molecule. An example of the CVs for bare and covered with DDT gold electrode is presented in Figure 2. First of all, it was established that in a row of 1, 4, 16, and 24 h, the immobilization of 16 h was enough to obtain the organic layer, effectively blocking the electrode surface (see Figure 2, for the electrode treated in the cyclic potential mode in NaOH). The results for 24 h fully coincided with those for 16 h. The results for 1 and 4 h were poorly reproduced. So, all the following experiments were conducted with an immobilization time of 16 h. Then, in the very first stages, it was found that the chemical reduction exhibited an awful reproducibility (a low repeatability of the chemical treatment was also reported, for example, by Ho et al. [56]), so we did not study this method further. The results on the surface coverage (in %) are presented in Table 2. The data for the electrode pretreated without the electrochemical stage are also presented.

It can be seen that without reductive electrochemical pretreatment, the surface coverage is relatively low (60 ± 10%). Among the pretreatments applied, the best coverage value and the higher reproducibility were shown by both cyclic and potentiostatic modes in PBS. Thus, to maximize the surface coverage and reproducibility, it was decided to consistently use these pretreatments, as shown in the 7th row in Table 1.

Thus, the electrochemical pretreatment providing a higher surface coverage with DDT was chosen. The next step was to approbate the chosen procedure for the formation of the DNA-aptamer layer.

#### 3.2.2. Biomolecules’ Immobilization Procedure Partial Optimization

**Figure 3:**
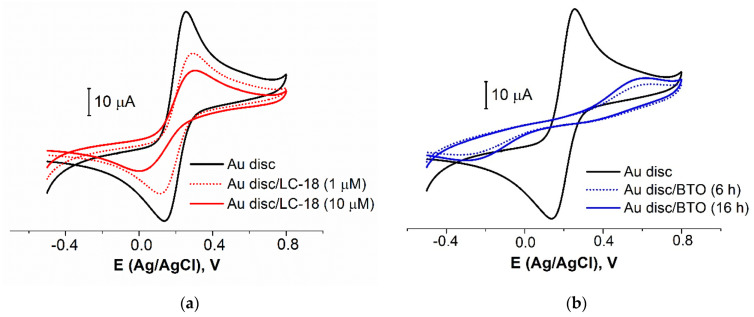
CVs for bare Au disc electrode and electrodes with LC-18 aptamer layer obtained from the solutions of different concentrations (**a**) and BTO layer obtained for different immobilization time (**b**). Solution: 0.025 M K_3/4_[Fe(CN)_6_] in PBS (7.4).

The tertiary structure of the aptamer LC-18, capable of binding to lung cancer biomarkers [41], was identified using small-angle X-ray scattering and molecular simulations [59]. The thiolated group was attached at the part of this aptamer that is not involved tertiary structure formation, so the aptamer’s active site appeared to be on the top of the attached molecule. The chosen pretreatment protocol was applied for the LC-18 aptamer immobilization from the solutions of two concentrations (1 and 10 µM). Expectedly (Figure 3a and Table 3), the higher concentration led to the higher surface coverage value (42 vs. 22%). Diaz-Amaya and co-authors [27] found an aptamer concentration of 10 µM to be optimal for the layer preparation. An additional blocking agent (BA) was necessary to increase the electrode surface blocking (as using a higher concentration of DNA-aptamer would increase the cost of the aptasensor).

A blocking agent is used to fill the electrode surface uncovered with the aptamer. Mercaptohexanol (MCH), mercaptoethanol (MET), bovine serum albumin (BSA) [60], commercial proteins [27], etc. [35], are quite widely used as BAs. However, large proteins may lead to a steric hindrance for the sensing aptamer layer (Figure 1), and short thiols form SAMs with a low stability under electrode potential change [51]. In addition, the different nature of the aptamer and BA molecules may result in an undesired lateral intermolecular interaction [61]. In this work, we suggest using a blocking thiolated oligonucleotide (BTO) with an unrelated sequence as the BA to avoid possible difficulties caused by the difference in aptamer and BA nature. So, BTO as the BA fills the defects in the aptamer layer. In addition, since BTO molecules are sufficiently short (23 nt), they did not hinder aptamer molecules from binding to the target object (Figure 1) because we chose a BTO with a molecule length equal to that of the linker used for aptamer binding with the electrode surface. Thus, the BTO layer would stay under the aptamer 3D structure so that aptamer molecules would bind the target protein without any steric hindrance. One more and crucial point is that the aptamer and BTO have the exact nature. This allows for avoiding repulsing or other undesired interactions between the recognizing and blocking molecules.

BA is typically immobilized when the aptamer layer has been formed. So, to decrease the time of contact of the prepared aptamer layer with the BTO solution, we estimated the surface coverage by BTO after 2, 4, 6, and 16 h of immobilization. An immobilization time less than 6 h has shown a low repeatability of the results. That is why we focused on 6 and 16 h of immobilization (Figure 3b and Table 3). It was found that 6 h provided relatively a high coverage value and good reproducibility. So, 6 h would be enough to fulfill all the possible gaps in the aptamer layer by the BTO molecules.

**Figure 4:**
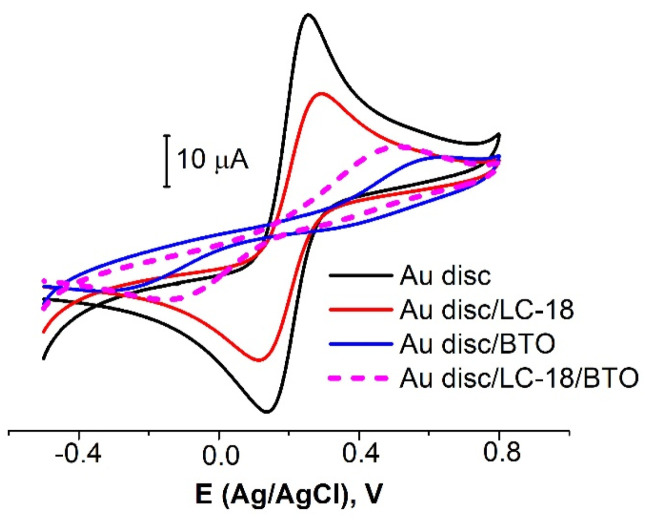
CVs for bare Au disc electrode (black curve) and electrodes with LC-18 aptamer layer (red curve), BTO layer (blue curve), and a layer of both aptamer and BTO (dashed curve). Solution: 0.025 M K_3/4_[Fe(CN)_6_] in PBS (7.4).

The consistent immobilization of LC-18 and BTO led to a surface coverage increase up to ≈70%, almost twice more extensive than for the aptamer alone (Figure 4, Table 3). As reported by Brothers with co-authors [35], denser SAMs are preferred for impedance-based biosensors. In our opinion, in the case under study, the modifying layer was dense enough to partially block the surface redox probes reactions, and it sparse enough to avoid steric hindrance for the aptamer molecules to bind target objects (tumor marker molecules). As the surface coverage under the chosen conditions exhibited good reproducibility, it was decided to use the above protocol to prepare an aptasensor.

#### 3.2.3. Biosensing Layer Stability Study

**Figure 5:**
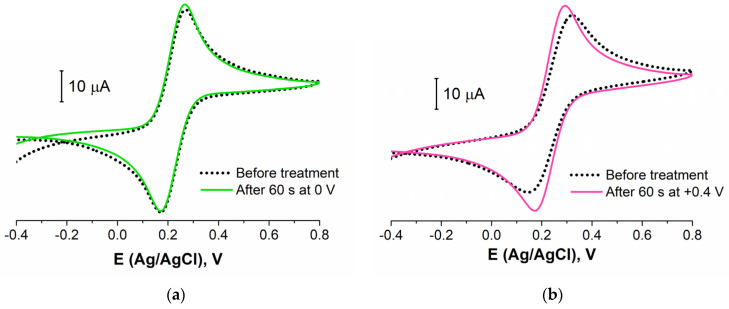

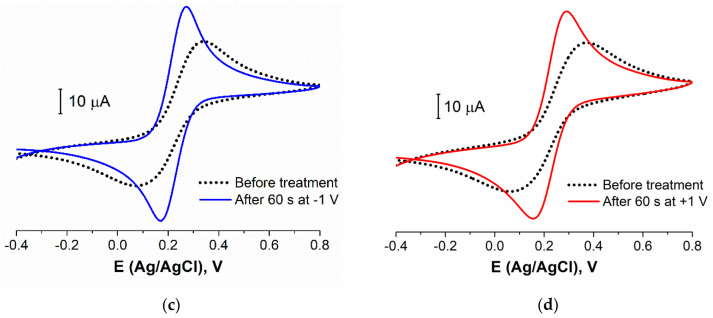
CVs for aptasensor (Au disc/LC-18) before (black dotted curve) and after (colored curves) potentiostatic treatment at: (**a**) 0 V; (**b**) +0.4 V; (**c**) −1 V; (**d**) +1 V. Solution: 0.025 M K_3/4_[Fe(CN)_6_] in PBS (7.4).

The stability of the layer of biomolecules was determined by its composition (different linkers used, additional BA, etc.) and structure (density, defects, etc.). To study the aptamer layer stability to the electrochemical influence, we carried out the experiments with a potentiostatic treatment of the aptasensor. The CVs and EIS data were registered before and after the electrode was kept for 60 s at a fixed potential. CVs for four different potential values of the treatment are presented in Figure 5. It is seen that the treatment at 0 V (Ag/AgCl) did not lead to the redox probe peaks’ change (Figure 5a). So, this potential was near the open circuit potential value and did not affect the layer structure and composition. The potential value of +0.4 V is an important point as this E is often achieved in the voltammetric methods (when CV, DPV, SWV, etc., are recorded). Figure 5b shows that 60 s at this potential value led to the partial destruction or reconstruction of the aptamer layer. The redox probe molecules achieved an electrode surface better than before (I increased by about 15%).

**Figure 6:**
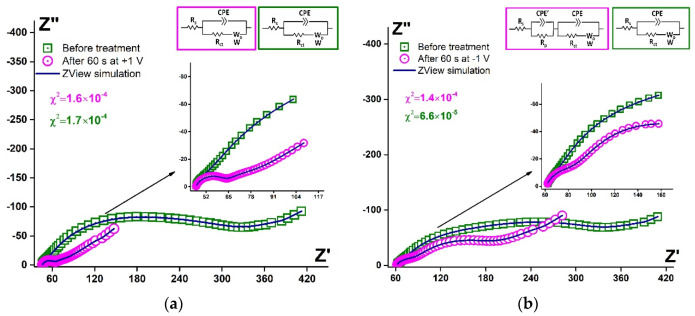
Simulated Nyquist plots (blue curves) and experimental data for Au disc/LC-18 before (green squares) and after (pink circles) potentiostatic treatment at (**a**) +1 V; (**b**) −1 V. Solution: 0.025 M K_3/4_[Fe(CN)_6_] in PBS (7.4). Insets: equivalent circuits.

**Figure 7:**
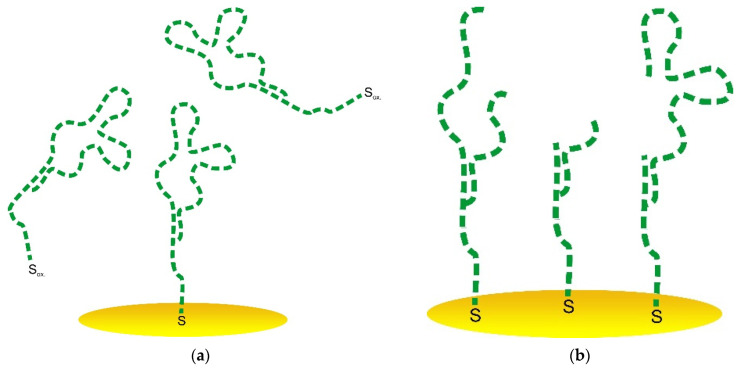
Schematic representation of the aptasensor surface with the partially desorbed aptamer (**a**) and partially decomposed aptamer (**b**).

It was reported by Ferapontova et al. [53] that the layer of DNA on the electrode surface exhibited an interval of stability between −0.6 and +0.6 V, and this region is limited to the reductive desorption and thiolates or guanine oxidation. According to Figure 5c,d, the treatment at higher potential values in cathodic and anodic regions led to the significant LC-18 aptamer layer destruction. EIS study was applied to reveal the possible mechanism of this process. It is seen (Figure 6) that layer destruction occurred differently when the electrode was exposed to positive or negative potentials. After 60 s at +1 V, the mechanism of the electrode process seemed to be unchanged and was still simulated with the modified Randles equivalent circuit. So, the aptamer may desorb from the electrode surface partially or even completely (Figure 7a). However, in the case of potentiostatic treatment at −1 V, the mechanism changed, giving an additional component in the circuit. The aptamer molecule decomposition might explain this with only partial desorption of the products from the surface (Figure 7b).

Fitting parameters for the EIS data simulation were calculated. The Warburg element exhibited non-ideal diffusion behavior for all the data obtained, and Rct decreased after any potentiostatic treatment. The parameters for CPE elements are presented in Table 4.

The parameter CPE-T decreased by one order of magnitude, both in the case of anodic and cathodic treatment. For the changed circuit, the CPE’-T was of the same order as the CPE-T before the cathodic potentiostatic treatment. So, after anodic treatment, the process of electron transfer seemed to be facilitated (time parameter decreased). Nevertheless, after cathodic treatment, the electron transfer phenomena in the studied system was divided into two processes, one of which was faster (facilitated, as well). The other one exhibited the same rate as before the layer deconstruction. So, this proved our previous suggestion that under cathodic potentiostatic treatment, the aptamer molecules decomposed, and only partial desorption of the products from the surface occurred.

Thus, the electrochemical stability of the LC-18 aptamer layer was studied under potentiostatic conditions. It was found that keeping the aptasensor at 0 V and +0.4 V (vs. Ag/AgCl) did not lead to the aptamer layer significant destruction. Nevertheless, higher potentials resulted in layer disruption. Presumably, anodic treatment led to complete aptamer desorption, while the cathodic potential influence resulted in aptamer molecule decomposition with partial desorption of the products.

#### 3.2.4. Biosensing Layer Binding Ability Testing

Generally, the electrochemical responses of the electrode with an aptamer layer in the presence and absence of the target are obtained and compared. Then, according to the results obtained, one of the common hypothetical mechanisms is suggested for the case [36]. Electron microscopic methods are used to evaluate the surface of the aptasensor, but only the state of the electrode itself is often studied (see, for example, [62]). Atomic force microscopy can be used to prove the formation of an aptamer layer and, possibly, its binding to target molecules (for example, [63]). However, the actual structure of the aptamer molecules and their changes upon binding with targets have not been studied yet for the majority of aptasensors. The suggested mechanisms are hypothetical and are proven indirectly via the changes in the electrochemical response, which, in turn, can be caused by other unaccounted factors. In the present work, more detailed studies of the mechanism of the formation of aptasensor’s electrochemical response were proposed.

In this work, to check whether the aptamer’s binding ability was not affected by the immobilization at the surface and to study the aptamer layer structure possible change after contact with the real samples (blood plasma), we suggested, for the first time, to apply the CLSM method [64,65].

The molecules of DNA aptamers consisted of adenine, guanine, cytosine, and thymine, containing heterocyclic fragments that exhibit weak fluorescence, usually in the near UV region of the spectrum [66]. The combinations of nucleotides in the aptamer allow one to expect weak autofluorescence of such macromolecules, both in the UV and visible ranges.

The LC-18 aptamer exhibited autofluorescence under excitation by a 405 nm laser. The emission maximum was fixed at 459 nm. So, using this fluorescent signal, the aptamer layer was visualized (in green) on the Au surface.

**Figure 8:**
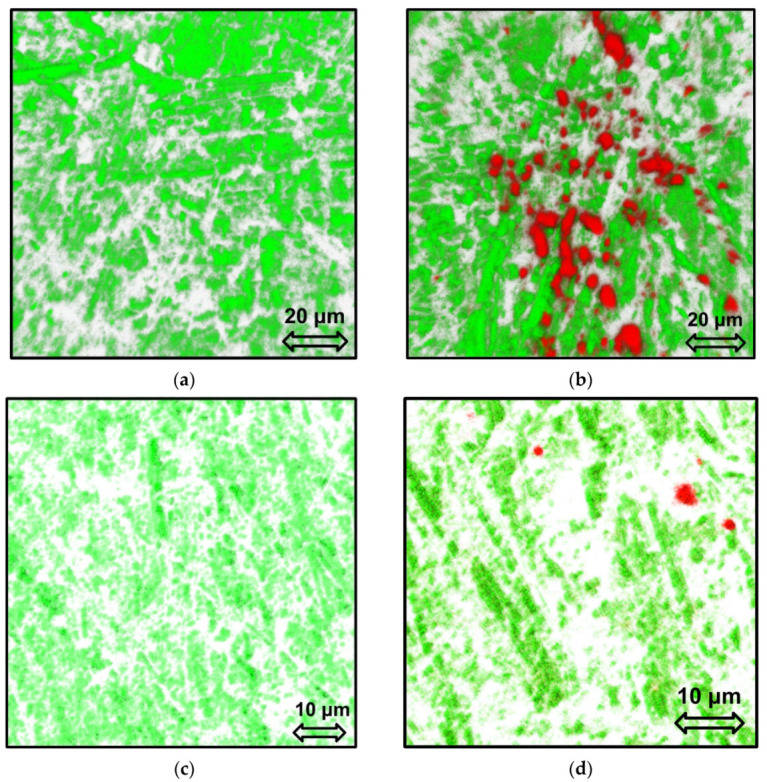
CLSM images of 3D reconstructed surface of aptasensor (Au disc/LC-18) with a dense (**a**,**b**) and a sparse (**c**,**d**) aptamer layer before (**a**,**c**) and after (**b**,**d**) contact with blood plasma from a lung cancer patient (**b**) and a healthy candidate (**d**). Green for the aptamer, red for proteins from blood plasma.

Figure 8a demonstrates the aptasensor surface with 70% aptamer coverage. It is seen that the electrode surface morphology affects the aptamer layer structure. After the aptasensor was kept in the patient’s blood plasma with lung cancer (LC group), the aptamer layer structure did not change (Figure 8b). In addition, plasma proteins visualized in red (the emission maximum for LC protein was found to locate at 560 nm) can be found on the surface of the aptamer layer. To determine the plasma proteins of the healthy candidate’s samples (H group), we had to prepare aptasensors with a lower surface coverage (about 55%, Figure 8c). Moreover, this allowed us to visualize the healthy plasma proteins (emission maximum at 500 nm) on the electrode surface uncovered with the aptamer (Figure 8d).

Thus, it can be concluded that the aptamer layer structure was not affected by being kept in the blood plasma samples, and the LC-18 aptamer retained its ability of selective binding of target molecules (proteins–tumor markers) after immobilization to the electrode surface. Moreover, the proteins from the H group blood plasma did not bind to the aptamer, covering only the electrode surface in the gaps of the aptamer layer.

In addition, we established whether the BTO layer could bind the target protein or the blood plasma (both the LC and H samples) and somehow affects its structure or properties. It was found that the electrochemical signal of the electrode covered with the BTO layer did not change after incubation with LC or H plasma samples (Figure S1, Supplementary Materials). Thus, the BTO layer effectively blocks the electrode surface from protein interaction, and it is not involved in capturing the protein from the plasma samples.

### 3.3. Useful Signal Determination

So, after the formation of the sensing layer, the next stage of the new aptasensor development is the study of its analytical performance. Differential-pulse voltammetry (DPV), electrochemical impedance spectroscopy (EIS), square-wave voltammetry (SWV), alternating current voltammetry (ACV), and other methods are used to detect the presence of the target objects. For this, the magnitude of the selected feature (peak of a current, semicircle part of a Nyquist plot, etc.) is compared in the response of the aptasensor before and after contact with the test sample. However, the entire electrochemical response is complex and is composed of many features. When a real object is analyzed (blood, urine, saliva, etc.), every sample component may affect the electrochemical response. So, it is necessary to extract the part of the response that belongs to the target object. Thus, in addition to the classic approach, the signal (an informative part of the response) can be either a nontrivial feature (for example, the potential for the current growth beginning), or not one feature, but their paired or tripled combination (for example, “peak height + peak area + peak potential”). So, in this work, we attempted to identify the informative part of the LC-18 aptasensor’s electrochemical response based on the experimental data array obtained for the real samples (blood plasma). The critical step was to reveal the connection between the presence of the target tumor marker in the blood plasma samples and the electrochemical responses of the aptasensor. We applied two approaches described below.

#### 3.3.1. Utilization of Statistical Learning Models

We supposed that any electrochemical response can be converted into a set of features (measured parameters’ values and their combinations). Moreover, those features allow one to detect the presence of a target object from the signal of the aptasensor. The signal might consist of one feature. Otherwise, the signal might be complex and composed of several features.

The CV and EIS measurements in the PBS solution and the redox probe solution were carried out for 10 samples of blood plasma of patients with lung cancer and 10 samples of healthy candidates (Figures S2 and S3, Supplementary Materials). CVs and EIS data obtained were automatically processed. The following base-features were determined from the CVs (base-features CV):

- *Ea* and *Ec*—potential of the anodic and cathodic peaks of the redox probe reactions, respectively;
- *Einit_a* (*Einit_c*)—the potential of the beginning of the peak;
- *ΔE* = |*Ea*−*Ec*|;
- *Ew_a* (*Ew_c*) and *Esh_a* (*Esh_c*)—are parameters of the wideness and the shape of the peak, respectively;
- *Ia* and *Ic*—current of the redox probe oxidation and reduction processes, respectively;
- *Iinit_a* (*Iinit_c*)—current of the beginning of the peak;
- *Sa* and *Sc*–area under the peaks;
- *R(I)* and *R(S)*—the ratio of peak currents (*Ia*/*Ic*) and the area under the peaks (*Sa*/*Sc*), respectively.

EIS data were represented as the Cole−Cole complex capacitance plots (C′″C” coordinates, Figure S3, Supplementary Materials) [55,67]. The following EIS base-features were obtained from these plots:

- *C_dl_*—EDL capacitance;
- *ω_min_*—the frequency at which a local minimum is observed;
- *C_min_*—active capacitance at ωmin;
- *ω_max_*—the frequency at which a local maximum is observed.

The features listed above were obtained from CVs and EIS data recorded for the aptasensor before and after incubation with the blood plasma sample. Then, the results obtained were used to calculate the following “change-features”:

(I) «Delta» (*Dlt*) was found according to the formula:

(1) $$Dlt\left[P\right]=\;P_{0}-P_{pl}$$

where *P_0_* is a feature’s value for aptasensor before contact with the blood plasma, and *P_pl_* is this feature’s value after incubation with the plasma sample for 1 h.

(II) The increment (*Incr*) of a feature was calculated as follows:

(2) $$Incr\left[P\right]=\frac{Dlt\left[P\right]}{P_{0}\;}\times 100\%$$

Thus, the dataset consisting of 12 base-features (eight for CVs and four for EIS results) and 24 change-features (*Dlt* and *Incr*) calculated for every base feature was marked to the samples’ groups “LC” (lung cancer) and “H” (healthy candidates). After that, we studied the dataset as a classification problem.

Using the traditional statistical approach, it was found that single features (base-features and change-features) did not allow for effective distinguishing between healthy candidates and LC patients. Thus, a “direct” CV and EIS data analysis did not provide a possibility to reveal tumor markers of lung cancer in the blood plasma. So, we moved to the analysis of the combinations of the features.

Several models of statistical learning were tried to predict the samples’ class. Based on the idea of interpretability, the set of models was narrowed to k-NN-classifiers (*n* = 1, 3, 5), linear SVMs (C = 0.1, 1, 10), and logistic regressions (C = 0.1, 1, 10). The aim of the models developed was to reveal which combinations of two or three features allow one to distinguish between samples of blood plasma obtained from healthy candidates and those obtained from patients with lung cancer. The quality of the models was evaluated as mean accuracy during leave-one-out cross-validation on the available data.

It was found that coupled combinations of parameters such as a shift in the position of the cathode peak and its shape change (*Dlt*[*Einit_c*] and *Incr*[*Esh_c*]), and a change in the cathodic current and an increase in the distance between the peaks (*Incr*[*Ic*] and *Dlt*[*ΔE*]) allowed one to distinguish the healthy and cancer samples with the mean accuracy of 0.70.

Using the triple combinations, it was possible to increase the mean accuracy up to 0.73. This result was provided by the following groups of features:

(1) *Incr[Ea]*, *Incr[Ic]*, *Dlt[Sa/Sc]*;

(2) *Incr[Ec]*, *Incr[Ew_c]*, *Dlt[Esh_c]*;

(3) *Incr[Ec]*, *Dlt[Ew_c]*, *Dlt[Esh_c]*;

(4) *Incr[Ic]*, *Dlt[Ea]*, *Dlt[Sa/Sc]*;

(5) *Incr[Sa/Sc]*, *Dlt[ΔE]*, *Dlt[Esh_c]*.

As can be seen, surprisingly, the cathodic peak, which follows the anodic one when the CV is registering, is more likely to point at the tumor marker present in the samples than the anodic peak. In addition, the ratio *Sa*/*Sc* is quite informative as well.

The (C′-C″) EIS base-features and change-features combinations did not distinguish between LC and H samples.

Thus, CV and EIS features one by one do not allow for distinguishing the blood plasma of a healthy candidate from the blood plasma of a patient with lung cancer. However, coupled and triple combinations of CV features allowed for approaching a maximal mean accuracy of 0.73. To further improve this index, a hypothetical mechanism of the processes on the electrode in the presence of tumor markers will be developed. The analytical conditions of the CVs registering will be optimized to increase the informativeness of the signals obtained. So far, it has been revealed that the cathodic peak of the hexacyanoferrate reduction is more sensitive to the presence of the tumor marker than the anodic one.

#### 3.3.2. EIS Data Simulation

Since the electrochemical signal recording is necessary to carry out at least twice—before and after contact of the aptasensor with the blood plasma—it is essential to minimize the external influence to preserve the sensing layer’s composition and structure. In addition, the Au surface can be damaged with redox probe ions, for example, CN—from [Fe(CN)_6_]^3^−^/4^−^^ [35,55]. So, the contact of the aptasensor with the redox probe solution and/or electrode polarization to the potential values far from the equilibrium (E_OC_) are better to be avoided in the analysis protocol. In this section, we provide the results of the analysis of the EIS data obtained in the background electrolyte at the potential near the E_OC_ for the aptasensor before and after incubation with the blood plasma samples. The impedance spectra were simulated, and the fitting parameters of the equivalent circuit were calculated.

**Figure 9:**
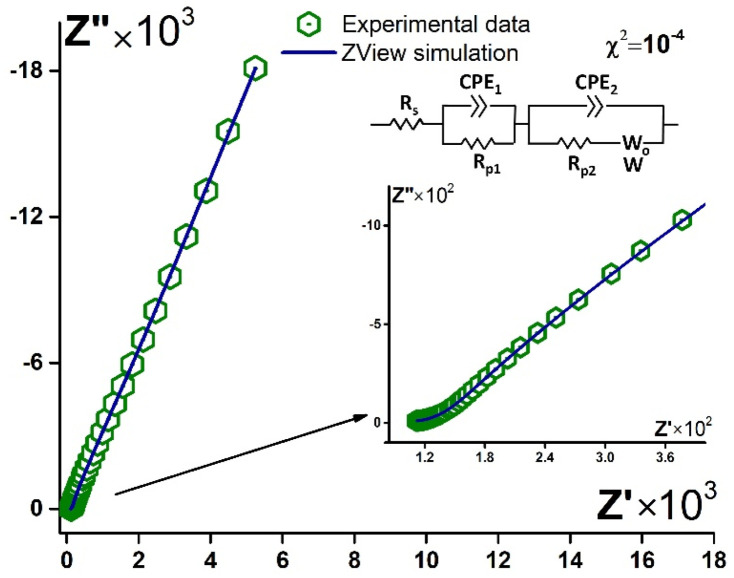
Typical experimental data (green hexagons) and simulated Nyquist plot (blue curve) for aptasensor (Au disc/LC-18) before/after contact with blood plasma. Solution: PBS (7.4). Inset: equivalent circuit.

It was revealed that before and after contact with the blood plasma, the spectra could be simulated with the same equivalent circuit. A typical Nyquist plot, experimental data, and simulation with the circuit are presented in Figure 9. Thus, importantly the mechanism of the processes in the electrode system did not change after the aptamer bonded the target molecule. Fitting parameters change. So, this is one more approval for the stability of the aptamer layer under contact with the biological liquid. Furthermore, the change of the parameters might point to the target tumor marker present in the samples.

Two main tendencies were revealed when H and LC blood plasma samples were compared. The *CPE_1_-T* parameter decreased by 1–2 orders of magnitude for LC samples and did not change for H blood plasma (Tables S1 and S2, Supplementary Materials). At the same time, the *CPE_2_-T* parameter increased after the binding of the tumor marker by the aptamer (LC samples) and decreased or did not change for the blood plasma of the H group. The values of *CPE-P* for all the fitted data were between 0.8 and 1 (Tables S1 and S2, Supplementary Materials), so the *CPE-T* parameter, in this case, was a characteristic of the capacitance.

We suggested that the first part of the circuit (*CPE_1_* and *Rp_1_*) described the process on the Au electrode surface, while the second part (*CPE_2_*, *Rp_2_*, *W*) belonged to the process in the aptamer layer. So, when aptamer bound the tumor marker, the double layer capacitance decreased at the electrode/solution interface and increased at the aptamer/solution boundary.

Thus, it was approved that the registration of EIS data in PBS and under E_OC_ conditions did not affect the aptamer layer state. In addition, the capacitive component might indicate the tumor marker’s presence in the sample. In further studies, it is necessary to optimize the measurement conditions to increase the informative part of the response even under such a small external impact on the system.

## 4. Conclusions

The reproducible and quite dense layer of the thiolated LC-18 DNA-aptamer specific to lung cancer tumor marker on the surface of the gold electrode was obtained. The stability of the composition, structure, and binding ability of the aptamer layer was studied. The potentiostatic treatment of the aptasensor at potentials of 0 and +0.4 V did not affect the significant layer decomposition, while potentials up to +1 and −1 V resulted in its disruption. Presumably, the anodic treatment led to the aptamer desorption, and cathodic potential resulted in aptamer molecules’ decomposition with partial desorption of the products. It was also found that the aptamer layer structure was not affected by contact with the blood plasma samples. Moreover, using the CLSM method, the ability of LC-18 aptamer to selectively bind the target molecules even after immobilization to the electrode surface was approved. At the same time, the proteins from the “healthy” blood plasma did not bind to the aptamer and were bound to the uncovered electrode surface.

In the present work, for the first time, the thiolated oligonucleotide with an unrelated sequence and the same length as a linker binding the aptamer with gold was suggested to block the uncovered electrode surface. It has an intimate nature to the aptamer molecules to avoid unwanted intermolecular interactions. So, this BA fills the defects in the aptamer layer and does not hinder aptamer molecules from binding to the target object.

The attempt to identify the informative part of the LC-18 aptasensor’s electrochemical response was made based on the experimental data array obtained for the real samples (blood plasma). Using statistical machine learning models, it was revealed that the features from CV and EIS data one by one did not allow for distinguishing the blood plasma of a healthy candidate from the blood plasma of a patient with lung cancer. At the same time, the combinations of CV features (coupled and tripled) allowed for approaching a mean accuracy of 0.73. In addition, this approach allowed us to obtain valuable information for further study of the mechanism of the aptasensor’s response formation.

Using ZView simulation, it was proven that non-Faradic EIS data collection did not affect the aptamer layer state. The capacitive component can indicate the tumor marker’s presence in the blood sample.

Thus, to further develop the LC-18 aptasensor for the lung cancer biomarker determination, it is necessary to optimize the measurement conditions for both methods (CV and non-Faradic EIS) to increase the informative part of the response.

## Figures and Tables

**Table 1:** Experiment planning matrix for choosing the method of the reductive pretreatment of gold electrodes at the last stage.

Protocol	Electrochemical Treatment					Chemical Reduction NaBH_4_ (1 m, 15 min)
	Cyclic Potential Mode (−1.4 to 0.8), 0.3 V/s		Potentiostatic Mode (−1.4 V), 30 s		Pulsed Mode (−1.4/0) V, 30 s, 3 min	
	NaOH (0.5 M)	PBS (7.4)	NaOH (0.5 M)	PBS (7.4)	PBS (7.4)	
1	×	–	–	–	–	–
2	–	×	–	–	–	–
3	–	–	×	–	–	–
4	–	–	–	×	–	–
5	–	–	–	–	×	–
6	–	–	–	–	–	×
7	–	×	–	×	–	–

**Table 2:** Surface coverage of the gold electrode with DDT (16 h of immobilization) after different electrochemical pretreatments were applied.

Electrochemical Treatment	Solution	DDT Coverage, %
Cyclic potential mode	NaOH	88 ± 12
	PBS	96 ± 2
Potentiostatic mode	NaOH	93 ± 2
	PBS	98 ± 2
Pulsed mode	PBS	90 ± 10
–	–	60 ± 10

**Table 3:** Surface coverage of the gold electrode with LC-18 and BTO under varying concentrations or immobilization times.

Modifier	Concentration, µM	Immobilization Time, h	Surface Coverage, %
LC-18	1	16	22 ± 6
	10		42 ± 2
BTO	10	6	89 ± 4
		16	86 ± 1
LC-18/BTO	10/10	16/6	68 ± 2

**Table 4:** EIS data fitting parameters for CPE for the aptasensor before and after cathodic and anodic potentiostatic treatment.

Parameter	Before Treatment	After 60 s at −1 V	After 60 s at +1 V
CPE-T, Ω^−1^ × s^α^	(4.2 ± 0.9) × 10^−5^	(3.1 ± 0.6) × 10^−6^	(1.9 ± 0.2) × 10^−6^
CPE-P (α)	0.72 ± 0.06	0.85 ± 0.04	0.96 ± 0.01
C_dl_, F	–	(2.6 ± 0.9) × 10^−3^	(1.0 ± 0.4) × 10^−3^
CPE’-T, Ω^−1^ × s^α^	–	(3.6 ± 0.9) × 10^−5^	–
CPE’-P (α)	–	0.86 ± 0.05	–
C’_dl_, F	–	(2.2 ± 0.7) × 10^−2^	–

## Data Availability

Data is contained within the article or Supplementary Materials.

## Supplementary Materials

The following are available online at https://www.mdpi.com/article/10.3390/s21237851/s1. Figure S1: CV data for bare Au disc electrode (black curve), BTO layer on its surface (blue curve), and Au disc/BTO after incubation with blood plasma of LC patient (pink curve) or healthy candidate (green curve). Solution: 0.025 M K3/4[Fe(CN)6] in PBS (7.4). Figure S2: CV data for the blood plasma samples of healthy candidates (a) and patients with lung cancer (b). Data analysis and visualization were performed using Anaconda Python 3.7 Distribution. Figure S3: EIS data for the blood plasma samples of healthy candidates (a,c) and patients with lung cancer (b,d). Data analysis and visualization were performed using Anaconda Python 3.7 Distribution. Table S1: EIS data fitting parameters for six different LC plasma samples. Table S2: EIS data fitting parameters for six different H plasma samples.

## References

[1] Akhtartavan S., Karimi M., Sattarahmady N., Heli H. (2019). An electrochemical signal-on apta-cyto-sensor for quantitation of circulating human MDA-MB-231 breast cancer cells by transduction of electro-deposited non-spherical nanoparticles of gold. J. Pharm. Biomed..

[2] Siller I., Preuss J., Urmann K., Hoffmann M., Scheper T., Bahnemann J. (2020). 3D-printed flow cells for aptamer-based impedimetric detection of E. coli crooks strain. Sensors.

[3] Sun X., Li C., Zhu Q., Chen J., Li J., Ding H., Sang F., Kong L., Chen Z., Wei Q. (2020). A novel ultrasensitive sandwich-type photoelectrochemical immunoassay for PSA detection based on dual inhibition effect of Au/MWCNTs nanohybrids on N-GQDs/CdS QDs dual sensitized urchin-like TiO_2_. Electrochim. Acta.

[4] Upan J., Banet P., Aubert P., Ounnunkad K., Jakmunee J. (2020). Sequential injection-differential pulse voltammetric immunosensor for hepatitis B surface antigen using the modified screen-printed carbon electrode. Electrochim. Acta.

[5] Figueroa-Miranda G., Wu C., Zhang Y., Nörbel L., Lo Y., Tanner J., Elling L., Offenhäusser A., Mayer D. (2020). Polyethylene glycol-mediated blocking and monolayer morphology of an electrochemical aptasensor for malaria biomarker detection in human serum. Bioelectrochemistry.

[6] Guo W., Zhang C., Ma T., Liu X., Chen Z., Li S., Deng Y. (2021). Advances in aptamer screening and aptasensors’ detection of heavy metal ions. J. Nanobiotechnol..

[7] Ding J., Zhang D., Liu Y., Yu M., Zhan X., Zhang D., Zhou P. (2019). An electrochemical aptasensor for detection of lead ions using a screen-printed carbon electrode modified with Au/polypyrrole composites and toluidine blue. Anal. Methods.

[8] Lee C., Yu S., Kim T. (2020). A “turn-on” electrochemical aptasensor for ultrasensitive detection of Cd^2+^ using duplexed aptamer switch on electrochemically reduced graphene oxide electrode. Microchem. J..

[9] Ni S., Qiao L., Shen Z., Gao Y., Liu G. (2019). Physical absorption vs covalent binding of graphene oxide on glassy carbon electrode towards a robust aptasensor for ratiometric electrochemical detection of vascular endothelial growth factor (VEGF) in serum. Electrochim. Acta.

[10] Ghanbari K., Roushani M., Soheyli E., Sahraei R. (2019). An electrochemical tyrosinamide aptasensor using a glassy carbon electrode modified by N-acetyl-L-cysteine-capped Ag-In-S QDs. Mater. Sci. Eng. C.

[11] Gliga L., Iacob B., Chesches B., Florea A., Barbu-Tudoran L., Bodoki E., Oprean R. (2020). Electrochemical platform for the detection of adenosine using a sandwich-structured molecularly imprinted polymer-based sensor. Electrochim. Acta.

[12] Zhang R., Liu L., Mao D., Luo D., Cao F., Chen Q., Chen J. (2020). Construction of electrochemical aptasensor of carcinoembryonic antigen based on toehold-aided DNA recycling signal amplification. Bioelectrochemistry.

[13] Khan R., Ben Aissa S., Sherazi T., Catanante G., Hayat A., Louis Marty J. (2019). Development of an impedimetric aptasensor for label free detection of patulin in apple juice. Molecules.

[14] Li Y., Liu D., Zhu C., Shen X., Liu Y., You T. (2020). Sensitivity programmable ratiometric electrochemical aptasensor based on signal engineering for the detection of aflatoxin B1 in peanut. J. Hazard. Mater..

[15] Liu J., Kong D., Liu Z., Liu H., Yi J., Tian D., Xia F., Zhou C. (2020). Three-dimensional mesoporous dendritic fibrous nanosilica as a highly efficient DNA amplification platform for ultrasensitive detection of chlorpyrifos residues. Sens. Actuators B-Chem..

[16] Baghayeri M., Alinezhad H., Fayazi M., Tarahomi M., Ghanei-Motlagh R., Maleki B. (2019). A novel electrochemical sensor based on a glassy carbon electrode modified with dendrimer functionalized magnetic graphene oxide for simultaneous determination of trace Pb(II) and Cd(II). Electrochim. Acta.

[17] Khosropour H., Rezaei B., Rezaei P., Ensafi A. (2020). Ultrasensitive voltammetric and impedimetric aptasensor for diazinon pesticide detection by VS 2 quantum dots-graphene nanoplatelets/carboxylated multiwalled carbon nanotubes as a new group nanocomposite for signal enrichment. Anal. Chim. Acta.

[18] Wei M., Yue S., Zhang W., Li X. (2020). Development of an electrochemical aptasensor using Au octahedra and graphene for signal amplification. Anal. Methods.

[19] An K., Lu X., Wang C., Qian J., Chen Q., Hao N., Wang K. (2019). Porous gold nanocages: High atom utilization for thiolated aptamer immobilization to well balance the simplicity, sensitivity, and cost of disposable aptasensors. Anal. Chim. Acta.

[20] Lu H., Wang G., Dai R., Ding X., Liu M., Sun H., Sun C., Zhao G. (2019). Visible-light-driven photoelectrochemical aptasensor based on reduced graphene oxide/Ti-Fe-O nanotube arrays for highly sensitive and selective determination of microcystin-LR. Electrochim. Acta.

[21] Bagheri H., Bakhsh R. (2019). Ultrasensitive and reusable electrochemical aptasensor for detectionof tryptophan using of [Fe(bpy)_3_](p-CH_3_C_6_H_4_SO_2_)_2_ as an electroactive indicator. J. Pharm. Biomed..

[22] An Y., Jin T., Zhu Y., Zhang F., He P. (2019). An ultrasensitive electrochemical aptasensor for the determination of tumor exosomes based on click chemistry. Biosens. Bioelectron..

[23] Abnous K., Mohammad Danesh N., Alinezhad Nameghi M., Ramezani M., Alibolandi M., Lavaee P., Mohammad Taghdisi S. (2019). An ultrasensitive electrochemical sensing method for detection of microcystin-LR based on infinity-shaped DNA structure using double aptamer and terminal deoxynucleotidyl transferase. Biosens. Bioelectron..

[24] Xie S., Zhang J., Teng L., Yuan W., Tang Y., Peng Q., Tang Q. (2019). Electrochemical detection of lipopolysaccharide based on rolling circle amplification assisted formation of copper nanoparticles for enhanced resistance generation. Sens. Actuators B-Chem..

[25] Bai Z., Chen Y., Li F., Zhou Y., Yin H., Ai S. (2019). Electrochemical aptasensor for sulfadimethoxine detection based on the triggered cleavage activity of nuclease P1 by aptamer-target complex. Talanta.

[26] Fatemi F. (2020). Design and fabrication of a label-free aptasensor for rapid and sensitive detection of endoglucanase. Int. J. Biol. Macromol..

[27] Diaz-Amaya S., Lin L., DiNino R., Ostos C., Stanciu L. (2019). Inkjet printed electrochemical aptasensor for detection of Hg^2+^ in organic solvents. Electrochim. Acta.

[28] Feng Y., Yan T., Wu T., Zhang N., Yang Q., Sun M., Yan L., Du B., Wei Q. (2019). A label-free photoelectrochemical aptasensing platform base on plasmon Au coupling with MOF-derived In_2_O_3_@g-C_3_N_4_ nanoarchitectures for tetracycline detection. Sens. Actuators B-Chem..

[29] Xu N., Hou T., Li F. (2019). A label-free photoelectrochemical aptasensor for facile and ultrasensitive mercury ion assay based on a solution-phase photoactive probe and exonuclease III-assisted amplification. Analyst.

[30] Esfahani S., Rouhani S., Ranjbar Z. (2019). Electrochemical solid-state nanosensor based on a dual amplification strategy for sensitive detection of (FeIII -dopamine). Electrochim. Acta.

[31] Liu M., Sun C., Wang G., Wang Y., Lu H., Shi H., Zhao G. (2018). A simple, supersensitive and highly selective electrochemical aptasensor for Microcystin-LR based on synergistic signal amplification strategy with graphene, DNase I enzyme and Au nanoparticles. Electrochim. Acta.

[32] Liu X., Tang Y., Liu P., Yang L., Li L., Zhang Q., Zhou Y., Hossain Khana Z. (2019). A highly sensitive electrochemical aptasensor for detection of microcystin-LR based on a dual signal amplification strategy. Analyst.

[33] Hongxia C., Zaijun L., Ruiyi L., Guangli W., Zhiguo G. (2019). Molecular machine and gold/graphene quantum dot hybrid based dual amplification strategy for voltammetric detection of VEGF165. Microchim. Acta.

[34] Sun Y., Jin H., Jiang X., Gui R. (2020). Black phosphorus nanosheets adhering to thionine-doped 2D MOF as a smart aptasensor enabling accurate capture and ratiometric electrochemical detection of target microRNA. Sens. Actuators B-Chem..

[35] Brothers M., Moore D., Lawrence M., Harris J., Joseph R., Ratcliff E., Ruiz O., Glavin N., Kim S. (2020). Impact of Self-Assembled Monolayer Design and Electrochemical Factors on Impedance-Based Biosensing. Sensors.

[36] De-Los-Santos-Alvarez N., Jesus Lobo-Castanon M., Miranda-Ordieres A., Tunon-Blanco P. (2008). Aptamers as recognition elements for label-free analytical devices. Trends Analyt. Chem..

[37] Negahdary M. (2020). Electrochemical aptasensors based on the gold nanostructures. Talanta.

[38] Zohreh M., Ghoreishi S., Behpour M., Mohammadhassan M. (2017). Applied electrochemical biosensor based on covalently self assembled monolayer at gold surface for determination of epinephrine in the presence of Ascorbic acid. Arab. J. Chem..

[39] Dutta G., Jo K., Lee H., Kim B., Woo H., Yang H. (2012). Time-dependent decrease in the enhanced electrocatalytic activities observed after three different pretreatments of gold electrodes. J. Electroanal. Chem..

[40] Zamay G., Kolovskaya O., Zamay T., Glazyrin Y., Krat A., Zubkova O., Spivak E., Wehbe M., Gargaun A., Muharemagic D. (2015). Aptamers selected to postoperative lung adenocarcinoma detect circulating tumor cells in human blood. Mol. Ther..

[41] Zamay G.S., Ivanchenko T., Zamay T.N., Grigorieva V., Glazyrin Y., Kolovskaya O., Garanzha I., Barinov A., Krat A., Mironov G. (2017). DNA-aptamers for characterization of histological structure of lung adenocarcinoma. Mol. Ther. Nucleic Acid..

[42] Zamay G.S., Zamay T.N., Kolovskii V.A., Shabanov A.V., Glazyrin Y.E., Veprintsev D.V., Krat A.V., Zamay S.S., Kolovskaya O.S., Gargaun A. (2016). Electrochemical aptasensor for lung cancer-related protein detection in crude blood plasma samples. Sci. Rep..

[43] Cole A.J., Clifton-Bligh R., Marsh D.J. (2015). Histone H2B monoubiquitination: Roles to play in human malignancy. Endocr. Relat. Cancer.

[44] Espinosa J.M. (2008). Histone H2B ubiquitination: The cancer connection. Genes Dev..

[45] Holterman D.A., Diaz J.I., Blackmore P.F., Davis J.W., Schellhammer P.F., Corica A., Semmes O.J., Vlahou A. (2006). Overexpression of alpha-defensin is associated with bladder cancer invasiveness. Urol. Oncol..

[46] Gaspar D., Freire J.M., Pacheco T.R., Barata J.T., Castanho M.A.R.B. (2015). Apoptotic human neutrophil peptide-1 antitumor activity revealed by cellular biomechanics. Biochim. Biophys. Acta.

[47] Mothes H., Melle C., Ernst G., Kaufmann R., von Eggeling F., Settmachera U. (2008). Human neutrophil peptides 1–3—early markers in development of colorectal adenomas and carcinomas. Dis. Markers.

[48] Bennett R.L., Bele A., Small E.C., Will C.M., Nabet B., Oyer J.A., Huang X., Ghosh R.P., Grzybowski A.T., Yu T. (2019). A mutation in histone H2B represents a new class of oncogenic driver. Cancer Discov..

[49] McKeown S.T.W., Lundy F.T., Nelson J., Lockhart D., Irwin C.R., Cowan C.G., Marley J.J. (2006). The cytotoxic effects of human neutrophil peptide-1 (HNP1) and lactoferrin on oral squamous cell carcinoma (OSCC) in vitro. Oral Oncol..

[50] Mi-Na N., Yang B., Hua-Li X., Hai-Tao L., Su-Lin X., Lu-Mei P., Prusky D. (2020). Modification performance and electrochemical characteristics of different groups of modified aptamers applied for label-free electrochemical impedimetric sensors. Food Chem..

[51] Miller C., Cuendet P., Gratzel M. (1991). Adsorbed hydroxy thiol monolayers on gold electrodes: Evidence for electron tunneling to redox species in solution. J. Phys. Chem..

[52] Dai J., Li Z., Jin J., Cheng J., Kong J., Bi S. (2008). Study of the solvent effect on the quality of dodecanethiol self-assembled monolayers on polycrystalline gold. J. Electroanal. Chem..

[53] Ferapontova E. (2017). Hybridization biosensors relying on electrical properties of nucleic acids. Electroanalysis.

[54] Kekedy-Nagy L., Shipovskov S., Ferapontova E. (2019). Electrocatalysis of ferricyanide reduction mediated by electron transfer through the DNA duplex: Kinetic analysis by thin layer voltammetry. Electrochim. Acta.

[55] Bertok T., Lorencova L., Chocholova E., Jane E., Vikartovska A., Kasak P., Tkac J. (2019). Electrochemical impedance spectroscopy based biosensors: Mechanistic principles, analytical examples and challenges towards commercialization for assays of protein cancer biomarkers. ChemElectroChem.

[56] Ho L., Limson J., Fogel R. (2019). Certain methods of electrode pretreatment create misleading responses in impedimetric aptamer biosensors. ACS Omega.

[57] Carvalhal R., Freire R., Kubota L. (2005). Polycrystalline gold electrodes: A comparative study of pretreatment procedures used for cleaning and thiol self-assembly monolayer formation. Electroanalysis.

[58] Tkac J., Davis J. (2008). An optimised electrode pretreatment for SAM formation on polycrystalline gold. J. Electroanal. Chem..

[59] Morozov D., Mironov V., Moryachkov R.V., Shchugoreva I.A., Artyushenko P.V., Zamay G.S., Kolovskaya O.S., Zamay T.N., Krat A.V., Molodenskiy D.S. (2021). The role of SAXS and molecular simulations in 3D structure elucidation of a DNA aptamer against lung cancer. Mol. Ther. Nucleic Acids.

[60] Wang H., Fang Y., Yuan P., Wang A., Luo X., Feng J. (2019). Construction of ultrasensitive label-free aptasensor for thrombin detection using palladium nanocones boosted electrochemiluminescence system. Electrochim. Acta.

[61] Li Z., Zhang L., Zeng S., Zhang M., Du E., Li B. (2014). Effect of surface pretreatment on self-assembly of thiol-modified DNA monolayers on gold electrode. J. Electroanal. Chem..

[62] Taghdisi S., Danesh N., Nameghi M., Ramezani M., Alibolandi M., Hassanzadeh-Khayat M., Emrani A., Abnous K. (2019). A novel electrochemical aptasensor based on nontarget-induced high accumulation of methylene blue on the surface of electrode for sensing of α-synuclein oligomer. Biosens. Bioelectron..

[63] Song J., Li S., Gao F., Wang Q., Lin Z. (2019). An in situ assembly strategy for the construction of a sensitive and reusable electrochemical aptasensor. Chem. Commun..

[64] Shabalina A., Sharko D., Lapin I. (2019). Visualization of the effectiveness of the surface blocking of electrochemical sensors using laser confocal microscopy. Proc. SPIE.

[65] Lapin I., Shabalina A., Svetlichnyi V., Kolovskaya O. (2018). Visualization of Nanoconstructions with DNA-Aptamers for Targeted Molecules Binding on the Surface of Screen-Printed Electrodes. Proc. SPIE.

[66] Gustavsonn T., Markovitsi D. (2021). Fundamentals of the Intrinsic DNA Fluorescence. Acc. Chem. Res..

[67] Formisano N. (2015). A study on the Optimisation of Electrochemical Impedance Spectroscopy Biosensors. Ph.D. Thesis.

