# Supplementary material for: Development of Electrochemical Aptasensor for Lung Cancer Diagnostics in Human Blood
Source: Sensors (Basel). 2021 Nov 25;21(23):7851. doi: 10.3390/s21237851 (PMC8659852; doi:10.3390/s21237851)
Supplement: Supplementary file 1 [file sensors-21-07851-s001.zip › sensors-1427552-supplementary.pdf]

Supplementary Material

# ***Development of Electrochemical Aptasensor for Lung Cancer Diagnostics in Human Blood***

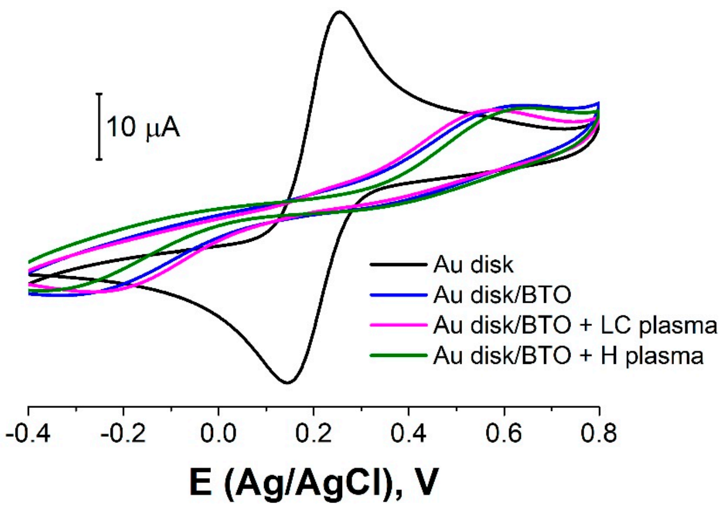


Figure S1 – CV data for bare Au disc electrode (black curve), BTO layer on its surface (blue curve), and Au disc/BTO after incubation with blood plasma of LC patient (pink curve) or healthy candidate (green curve). Solution: 0.025 M $K_{3/4}[Fe(CN)_6]$ in PBS (7.4).

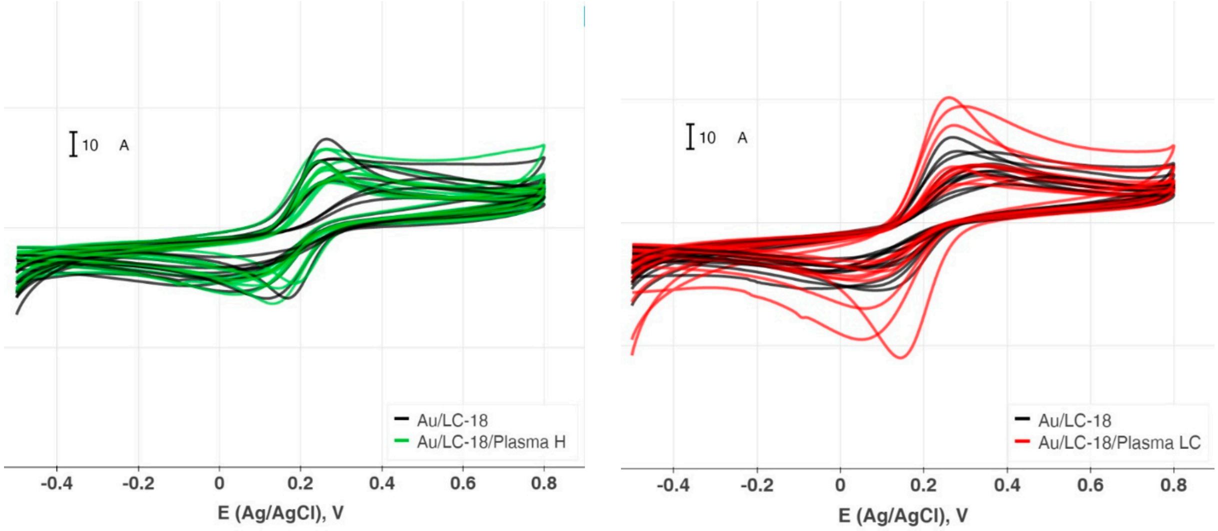


**a** **b**

Figure S2 – CV data for the blood plasma samples of healthy candidates (a) and patients with lung cancer (b). Data analysis and visualization was performed using Anaconda Python 3.7 Distribution.

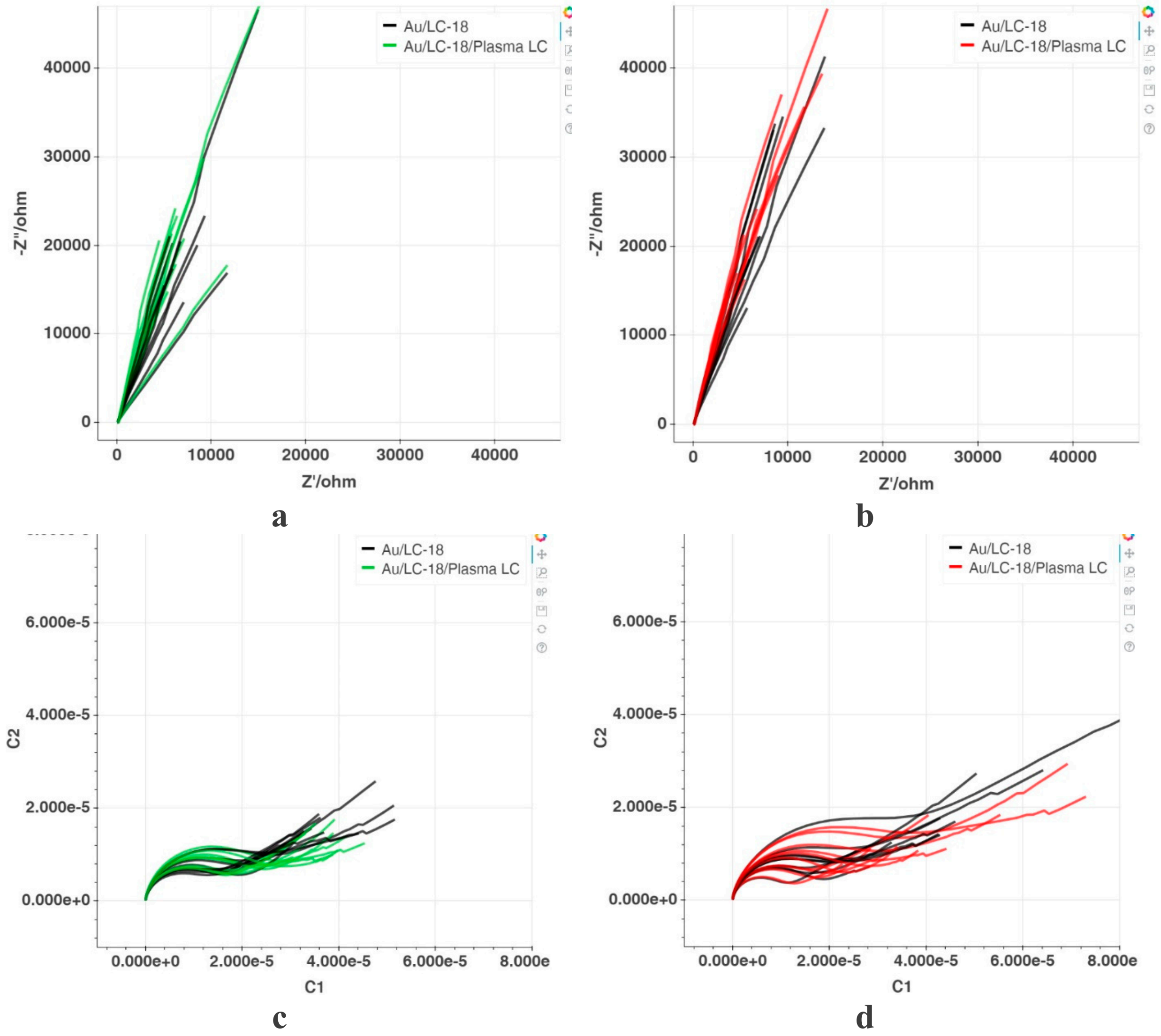


Figure S3 – EIS data for the blood plasma samples of healthy candidates (a, c) and patients with lung cancer (b, d). Data analysis and visualization was performed using Anaconda Python 3.7 Distribution.

Table S1 – EIS data fitting parameters for six different LC plasma samples.

| Parameter | Before | After | Before | After | Before | After | Before | After | Before | After | Before | After |
|---|---|---|---|---|---|---|---|---|---|---|---|---|
| $\chi^2$ | $2.7\times10^{-4}$ | $2.0\times10^{-4}$ | $5.2\times10^{-4}$ | $4.9\times10^{-4}$ | $4.4\times10^{-4}$ | $8.6\times10^{-5}$ | $2.2\times10^{-4}$ | $7.7\times10^{-4}$ | $3.9\times10^{-4}$ | $8.6\times10^{-4}$ | $8.1\times10^{-4}$ | $1.8\times10^{-4}$ |
| $R_{s1}$ | 88.1 | 92.7 | 64.4 | 60.5 | 80.3 | 67.2 | 54.5 | 103.8 | 108.0 | 71.3 | 116.0 | 93.3 |
| $CPE_1$-T | $1.1\times10^{-5}$ | $2.2\times10^{-7}$ | $2.4\times10^{-5}$ | $6.1\times10^{-6}$ | $1.3\times10^{-5}$ | $8.5\times10^{-6}$ | $1.2\times10^{-5}$ | $8.6\times10^{-7}$ | $1.1\times10^{-5}$ | $1.6\times10^{-7}$ | $4.7\times10^{-6}$ | $3.8\times10^{-7}$ |
| $CPE_1$-P | 0.82 | 0.89 | 0.81 | 0.81 | 0.81 | 0.92 | 0.80 | 0.93 | 0.82 | 0.80 | 0.82 | 0.80 |
| $R_{p1}$ | 18.1 | 21.9 | 29.0 | 38.1 | 48.2 | 19.9 | 24.5 | 28.2 | 21.8 | 51.4 | 29.3 | 45.7 |
| $CPE_2$-T | $7.3\times10^{-7}$ | $4.4\times10^{-6}$ | $1.7\times10^{-6}$ | $1.4\times10^{-5}$ | $1.2\times10^{-6}$ | $4.2\times10^{-5}$ | $5.9\times10^{-8}$ | $5.8\times10^{-6}$ | $1.2\times10^{-5}$ | $1.1\times10^{-6}$ | $9.7\times10^{-7}$ | $1.3\times10^{-6}$ |
| $CPE_2$-P | 0.82 | 0.81 | 0.86 | 0.82 | 0.80 | 0.81 | 0.82 | 0.82 | 0.80 | 1.0 | 0.80 | 0.99 |
| $R_{p2}$ | 24.7 | 105.7 | 38.1 | 33.9 | 39.3 | 19.7 | 47.2 | 90.8 | 11.6 | 48.2 | 10.9 | 63.2 |
| $W_2$-R | 2.71 | 1.27 | 1.07 | 1.15 | 1.06 | 0.98 | 1.88 | 1.98 | 1.21 | 1.23 | 1.29 | 1.59 |
| $W_2$-T | $1.7\times10^{-6}$ | $3.9\times10^{-7}$ | $2.2\times10^{-6}$ | $1.9\times10^{-6}$ | $4.1\times10^{-6}$ | $1.3\times10^{-6}$ | $5.1\times10^{-7}$ | $1.8\times10^{-9}$ | $9.5\times10^{-5}$ | $6.9\times10^{-7}$ | $1.5\times10^{-6}$ | $1.1\times10^{-6}$ |
| $W_2$-P | 0.41 | 0.43 | 0.43 | 0.78 | 0.64 | 0.43 | 0.35 | 0.26 | 0.81 | 0.41 | 0.53 | 0.41 |

Table S2 – EIS data fitting parameters for six different H plasma samples.

| Parameter | Before | After | Before | After | Before | After | Before | After | Before | After | Before | After |
|---|---|---|---|---|---|---|---|---|---|---|---|---|
| $\chi^2$ | $9.8\times10^{-4}$ | $8.4\times10^{-4}$ | $5.6\times10^{-5}$ | $1.9\times10^{-4}$ | $1.2\times10^{-4}$ | $8.5\times10^{-5}$ | $5.3\times10^{-5}$ | $4.6\times10^{-5}$ | $7.0\times10^{-4}$ | $8.2\times10^{-4}$ | $4.2\times10^{-4}$ | $9.8\times10^{-4}$ |
| $R_{s1}$ | 86.51 | 113.3 | 96.93 | 109.6 | 78.82 | 70.92 | 60.28 | 38.48 | 84.18 | 87.63 | 113.6 | 112 |
| $CPE_1$-T | $7.1\times10^{-6}$ | $3.9\times10^{-6}$ | $1.1\times10^{-6}$ | $1.7\times10^{-6}$ | $6.6\times10^{-6}$ | $8.3\times10^{-6}$ | $5.9\times10^{-6}$ | $7.2\times10^{-6}$ | $1.3\times10^{-6}$ | $4.9\times10^{-6}$ | $1.8\times10^{-6}$ | $8.1\times10^{-6}$ |
| $CPE_1$-P | 0.86 | 0.83 | 1.0 | 0.81 | 0.86 | 0.82 | 0.88 | 0.85 | 0.81 | 0.80 | 0.81 | 0.81 |
| $R_{p1}$ | 35.6 | 32.8 | 11.3 | 28.7 | 40.5 | 49.0 | 27.2 | 30.4 | 80.7 | 38.6 | 90.8 | 28.5 |
| $CPE_2$-T | $1.1\times10^{-5}$ | $7.5\times10^{-8}$ | $3.8\times10^{-7}$ | $6.2\times10^{-7}$ | $2.2\times10^{-5}$ | $2.1\times10^{-5}$ | $1.1\times10^{-5}$ | $8.4\times10^{-6}$ | $4.7\times10^{-6}$ | $5.9\times10^{-6}$ | $2.9\times10^{-6}$ | $9.2\times10^{-6}$ |
| $CPE_2$-P | 0.79 | 0.88 | 0.81 | 0.99 | 0.52 | 0.50 | 0.46 | 0.44 | 0.80 | 0.91 | 0.80 | 0.86 |
| $R_{p2}$ | 24.4 | 5.9 | 30.1 | 159.3 | 47.6 | 43.0 | 92.6 | 119.6 | 24.9 | 95.9 | 44.7 | 27.5 |
| $W_2$-R | 1.36 | 1.01 | 1.78 | 3.37 | 1.19 | 1.12 | 3.03 | 3.43 | 1.01 | 1.61 | 1.48 | 1.91 |
| $W_2$-T | $1.3\times10^{-4}$ | $1.9\times10^{-6}$ | $4.3\times10^{-7}$ | $4.4\times10^{-7}$ | $2.6\times10^{-6}$ | $2.9\times10^{-6}$ | $2.9\times10^{-6}$ | $3.4\times10^{-6}$ | $1.2\times10^{-5}$ | $1.8\times10^{-9}$ | $1.9\times10^{-6}$ | $3.5\times10^{-6}$ |
| $W_2$-P | 0.82 | 0.44 | 0.40 | 0.38 | 0.49 | 0.48 | 0.46 | 0.45 | 0.49 | 0.27 | 0.44 | 0.67 |
